# Roles of Al and Mg on the Microstructure and Corrosion Resistance of Zn-Al-Mg Hot-Dipped Coated Steel

**DOI:** 10.3390/ma17071512

**Published:** 2024-03-27

**Authors:** Taixiong Guo, Yuhao Wang, Liusi Yu, Yongqing Jin, Bitao Zeng, Baojie Dou, Xiaoling Liu, Xiuzhou Lin

**Affiliations:** 1 State Key Laboratory of Vanadium and Titanium Resources Comprehensive Utilization, Pangang Group Research Institute Co., Ltd., Panzhihua 617000, China; guotaixiong@126.com; 2 School of Materials Science and Engineering, Sichuan University of Science & Engineering, Zigong 643000, China; wangyuhao202206@163.com (Y.W.);; 3 Pangang Group Panzhihua Steel and Vanadium Co., Ltd., Panzhihua 617017, China; 4 Material Corrosion and Protection Key Laboratory of Sichuan Province, Sichuan University of Science & Engineering, Zigong 643000, China; 5 Zigong Ligong Technic Co., Ltd., Zigong 643000, China

**Keywords:** Zn-Al-Mg coating, microstructure, electrochemical corrosion, SVET, corrosion mechanism

## Abstract

In this work, a novel zinc–aluminum–magnesium (Zn-Al-Mg, ZM) coated steel was prepared using the hot-dip method. The microstructure and corrosion resistance of the ZM-coated steel were investigated. Compared to the conventional galvanized steel (GI), the ZM coating demonstrated a distinctive phase structure, consisting of Zn phase, binary eutectic (Zn/MgZn_2_), and ternary eutectic (Zn/Al/MgZn_2_). The corrosion resistance of the ZM-coated and GI-coated steels was evaluated by neutral salt spray test (NSST), polarization and electrochemical impedance spectroscopy (EIS). The results indicated that ZM-coated steel provided superior long-term corrosion protection in a NaCl environment compared to GI-coated steel. The scanning vibrating electrode technique (SVET) proved to be an effective method for investigating the evolution of the anodic and cathodic on the local coating surface. GI-coated steel exhibited a potential and current density distribution between the cathodic and anodic sites nearly three orders of magnitude higher than that of ZM-coated steel, suggesting a higher corrosion rate for GI-coated steel.

## 1. Introduction

Hot-dip zinc (GI) coating is an economically and environmentally friendly anti-corrosion coating that has been widely used for steel protection due to its barrier and galvanic protection [1,2,3]. However, it poses challenges in meeting the requirements for corrosion protection, particularly in harsh corrosion environments. Recently, new types of hot-dip alloy coatings have been developed, such as zinc–aluminum (Zn-Al, ZA) coating [2,3], zinc–aluminum–magnesium (Zn-Al-Mg, ZM) coating [4,5,6,7], by adding the Al, Mg elements to the molten Zn bath. This process may introduce new phases that can influence the composition and structure of the coatings, thereby affecting the corrosion performance. Therefore, the establishment of the relationship between the structure and corrosion performance is crucial for designing coated steel.

The elements employed in the galvanization process play an important role in producing uniform and exceptional galvanized steel. Normally, a small amount of Al (0.1–3 at.%) is introduced to the molten bath during the galvanization process. This results in the formation of an intermetallic film between the zinc coating and the steel substrate, which helps prevent the formation of Fe-Zn intermetallic phase or “outbursts” that could negatively impact the mechanical properties of the final coating [8,9,10]. Dou et al. developed a novel AESEC-gravimetric method to detect the dissolution process of galvanized steel in an HCl solution. Their research reveals the dissolution kinetics of galvanized steel and identifies the presence of an intermetallic layer in the form of Fe_2_Al_5_, with a total thickness of 50 nm [11].

Recently, zinc–aluminum–magnesium (Zn-Al-Mg) coatings have been innovated through hot-dip plating technology, which has attracted more and more attention due to its excellent corrosion resistance and self-healing capabilities [4,5,12,13,14]. Previous studies have indicated that the addition of Mg and Al elements to the molten bath undergoes eutectic reactions, resulting in the generation of various alloy phases such as MgZn_2_, which may form the Zn/MgZn_2_ binary eutectic phase or Zn/Al/MgZn_2_ ternary eutectic phase. These alloy phases, or the eutectic phase of zinc, exhibit preferential corrosion, reducing the dissolution rate of zinc, and thereby improving the corrosion resistance of the coating [4,15,16,17]. The amount of Mg and Al added into the molten bath also affects the structure and corrosion resistance. Mg, in particular, enhances corrosion resistance and offers a degree of self-healing performance [18]. However, the mass fraction of Mg cannot exceed 3%, as it may lead to a gradual deterioration of the coating’s corrosion resistance [19]. In contrast, the range of Al addition in Zn-Al-Mg coating is relatively wide and classified into three types: low aluminum (1~3 wt.%), medium aluminum (5~11 wt.%), and high aluminum (50~55 wt.%) [15].

Various types of Zn-Al-Mg coatings were developed by LeBozec et al. [4]. The corrosion performance of these coated steel was evaluated in an outdoor marine atmosphere for up to 2 years. It was found that fine microstructures enriched in eutectic phases exhibited the highest corrosion resistance. This was attributed to the smaller size of cathodic areas on the surface (zinc dendrites), the preferential dissolution of the Mg-rich phase (eutectic) and the formation of a stable-layered double hydroxide on the surface. Lee et al. [5] investigated the cut-edge corrosion behaviors of Zn-Al-Mg coated steel. The results indicated that the MgZn_2_ phase dissolved preferentially, leading to the co-precipitation of Mg(OH)_2_, Zn_5_(CO_3_)_2_(OH)_6_, and Zn_5_(OH)_8_Cl_2_∙H_2_O. This co-precipitation process enhanced the corrosion resistance of the Zn-Al-Mg coated steel. The corrosion resistance of Zn-Al-Mg coated steel is closely related to its phase composition, which affects the electrochemical behavior between the new phase and matrix. Therefore, monitoring the evolution of the electrochemical reaction is essential. The scanning vibrating electrode technique (SVET) is a relatively new technique that can distinguish the anodic and cathodic sites on the coating surface. This technique provides electrochemical information such as potential and current, and has been used in interpreting the corrosion mechanisms in various alloys [20,21].

Herein, a new ZM-coated steel was designed using the hot-dip method, with the addition of Mg and Al elements to the molten bath. The microstructure of the resulting ZM coating was characterized. The corrosion resistance of the ZM-coated steel will be investigated using electrochemical and neutral salt spray. The SVET technique will be employed to monitor the distribution of potential and current density on the coating surface, attempting to establish a connection between the structure and corrosion performance of the galvanized steel, and consequently provide valuable insights for the design of the galvanized steels.

## 2. Materials and Methods

### 2.1. Materials

An industrial hot-dip method was used to create ZM-coated steel samples. First, the mild steel (0.04 C, 0.03 Si, 0.20 Mn, 0.01 P, 0.01 S, and balance Fe in mass %) substrate (Shanghai Casting Enterprise Industrial Co., Ltd., Shanghai, China) was cleaned by degreasing, acid pickling, rinsing, and drying. After that, it was annealed at 780 °C for 60 s and subsequently cooled down to 450 °C. The heating and cooling rates were set at 10 °C per second. Following that, the coupons were immersed in a molten bath consisting of Zn-2.0% Al-1.5% Mg (Al: 2 wt.%, Mg: 1.5 wt.%) for 3 s at 440 °C. N_2_ gas (95% N_2_ + 5% H_2_) was utilized to protect the coupons throughout the annealing and hot-dip processes, enabling the Zn-Al-Mg (ZM) coated steel to be generated. Conventional hot-dip galvanized steel (GI) was also employed as a comparison in this work.

### 2.2. Corrosion Tests

The samples were subjected to neutral salt spray for atmospheric corrosion following ISO 9227: 2006 [22]. Deionized water and anhydrous ethanol were used to clean the samples. A 5 wt.% NaCl solution, adjusted to a pH of 6.5 to 7.2 by NaOH, was sprayed into the chamber at a constant temperature of 35 ± 2 °C, as indicated in the standard. The test samples were put at a 20° angle to the vertical direction on a plastic sample holder. The entire test lasted 40 days. In each test, three specimens were used to represent each substance. After different corrosion intervals, the corrosion morphologies of ZM- and GI-coated steel were photographed, the mass loss was weighted, and the weight loss rate was calculated as follows:

(1) $$v_{p}=8.76\frac{w_{1}-w_{0}}{\rho St}$$

where $v_{p}$ represents the weight loss rate (mm∙a^−1^), $w_{1}$ is the weight after removing corrosion products (g), w_0_ refers to the initial weight (g), ρ is the density (g∙cm^−3^), S corresponds to the exposed area (m^2^), t is the testing time (h).

Potentiodynamic polarization curves (PDP) and electrochemical impedance spectroscopy (EIS) were used to evaluate the corrosion performance of the GI- and ZM-coated steel in 3.5 wt.% NaCl solution at room temperature by a Potentiostat (AUTOLAB, Geneva, Switzerland), with a conventional three-electrode cell, platinum plate as the counter electrode, a saturated calomel electrode (SCE) as the reference electrode, and the GI- and ZM-coated steel as the working electrode. PDP test was obtained from −0.3 V (vs. OCP) to +0.3 V (vs. OCP) with a scan rate of 1 mV∙s^−1^. EIS test was performed at frequencies ranging from 100 kHz to 10 mHz by using a 10 mV amplitude sinusoidal voltage at OCP. The data were fitted using ZSimpWin 3.5 software. The PDP and EIS measurements were carried out three times to ensure the repeatability of the measurements.

Scanning vibrating electrode technique (SVET) was employed to monitor the localized corrosion potentials of GI- and ZM-coated steel immersed in 3.5 wt.% NaCl for various times. The SVET instrumentation was manufactured by Princeton (MA, USA) and controlled by dedicated software. The microelectrode had a platinum tip with a diameter of 20 μm and vibrated with an amplitude of 20 μm, positioned 200 μm away from the sample. A calibration process was performed to convert the measured potentials into current density at the corroding surface.

### 2.3. Characterization

The thickness and average coating weight per side of ZM- and GI-coated steels were obtained using a thickness tester and weight method. The surface morphology, cross-section microstructure, and chemical composition of ZM- and GI-coated steels were characterized by a scanning electron microscope (SEM, S4800, Hitachi, Tokyo, Japan) equipped with energy disperse spectroscopy (EDS, X-MaxN, Oxford, UK).

After the neutral salt spray test (NSST) and the electrochemical corrosion test, the corroded GI- and ZM-coated steel were characterized using X-ray diffraction (XRD, D8 ADVANCE, BRUKER, Ettlingen, Germany). The corrosion morphology and the composition of the coated steel were also analyzed by SEM and EDS.

## 3. Results

### 3.1. Microstructure of ZM- and GI-Coated Steel

Table 1 summarizes the thickness and average coating weight per side of ZM and GI coatings. The thickness of ZM and GI coating were 13.7 μm and 16.1 μm, respectively, with an average coating weight per side of 87 g∙m^−2^ and 104 g∙m^−2^.

**Figure 1:**
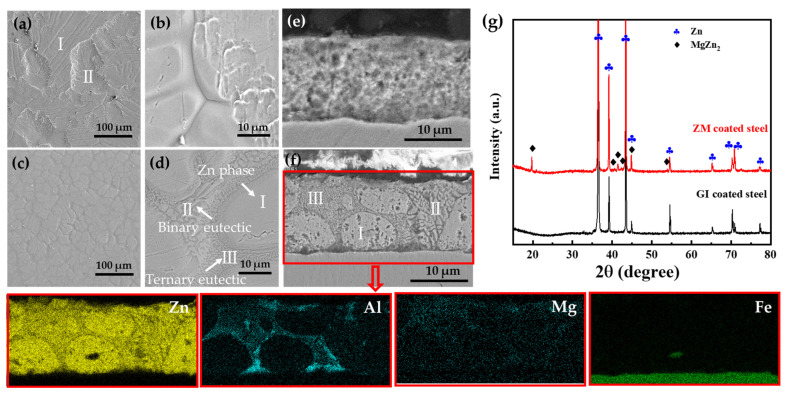
Surface micrographs of GI-coated steel (**a**,**b**), ZM-coated steel (**c**,**d**), the cross-section of GI-coated steel (**e**), ZM-coated steel (**f**), and XRD spectra of GI- and ZM-coated steel (**g**).

The surface morphology, cross-section, and phase structure of ZM- and GI-coated steels are presented in Figure 1. The chemical compositions of ZM- and GI-coated steels are summarized in Table 2. The surface of the GI coating exhibits two distinct morphologies: plate and dimple areas, as shown in Figure 1a,b. Both of these areas were zinc-enriched phases, as indicated by the composition of GI coating in Table 2. However, the formed ZM coating displays a new phase structure, consisting of enriched Zn phase, binary eutectic (Zn/MgZn_2_), and ternary eutectic (Zn/Al/MgZn_2_), when 2 wt.% Al and 1.5 wt.% Mg are added to the molten bath during the galvanized processing, as shown in Figure 1c–f [23,24,25]. The XRD spectra also illustrate the formation of a new phase (MgZn_2_) on the ZM-coated steel. The formation and distribution of this new phase in the enrichment Zn coating will lead to a change in the potential distribution on the surface of the ZM coating, thereby affecting its corrosion performance. Consequently, the corrosion performance of the ZM-coated steel was investigated in the subsequent session.

### 3.2. ZM- and GI-Coated Steel in NSST

#### 3.2.1. Optical Photographs of the Coated Steel

**Figure 2:**
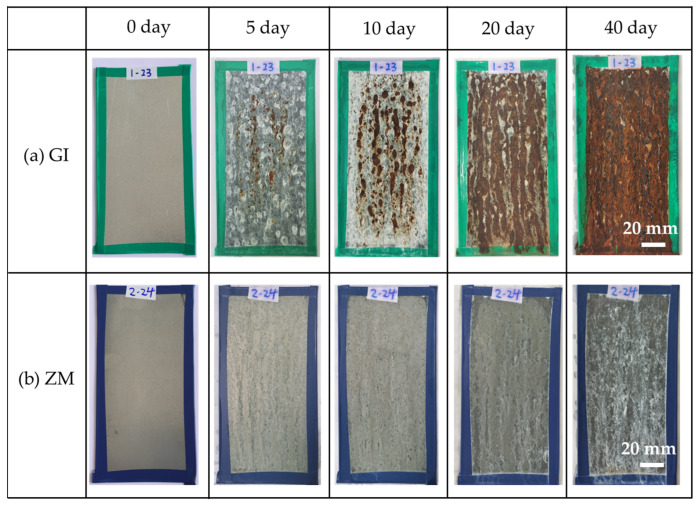
The photographs of (**a**) GI- and (**b**) ZM-coated steel after different times of NSST testing.

The photographs of ZM- and GI-coated steel before and after NSST for different exposed times are shown in Figure 2. The appearance of ZM- and GI-coated steel after the salt spray test is notably distinct, highlighting the difference in corrosion progress [12]. From Figure 2, it can be observed that red rust emerged on the surface of GI-coated steel after 5 days of salt spray testing and rapidly spread across the sample after 20 days of salt spray testing. In contrast, ZM-coated steel only exhibited slight white rust after 20 days of salt spray testing, with no presence of red rust throughout the entire period, indicating considerably slower corrosion progress. The visual inspection result demonstrated a significant difference between ZM- and GI-coated steel, suggesting an enhanced corrosion resistance of the ZM coating in NSST compared to the GI coating. These findings align with a previous study [26].

#### 3.2.2. Weight Loss Rate of the Coated Steel

**Figure 3:**
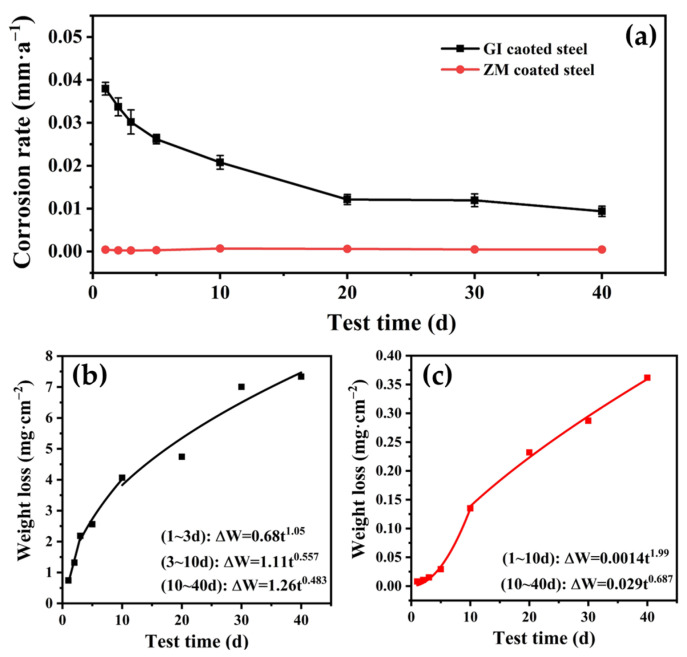
The weight loss rate of GI- and ZM-coated steel (**a**) and the weight loss of GI- (**b**) and ZM- (**c**) coated steel after different times of NSST experiments.

The weight loss rate of GI- and ZM-coated steel was calculated using Equation (1) and is depicted in Figure 3a based on NSST measurements conducted during different testing periods. The weight loss rate of GI-coated steel gradually decreased over time and reached a nearly steady state after 20 days of salt spraying. After a 40-day salt spray test, the weight loss rate of GI-coated steel was determined to be 0.0094 mm/year. In contrast, the weight loss rate of ZM-coated steel remained consistently low and stable throughout the exposed periods, measuring 0.00046 mm/year after a 40-day salt spray test.

It has been demonstrated in previous studies that the relationship between the weight loss of many metals and testing time follows the power function law, as depicted in Equation (2):

(2) $$∆W=At^{n}$$

where ∆W represents the weight loss (g/cm^2^), A is a constant, t is the testing time and n is a coefficient that reflects the character of atmospheric corrosion kinetics. In other words, if n > 1, it indicates a corrosion acceleration process, if n < 1, it denotes a corrosion deceleration process, and if n = 1, it signifies that the corrosion is constant [27,28,29].

For GI-coated steel (Figure 3b), the corrosion process can be described in three stages, in the initial period of exposure (1 to 3 days), the value of n is more than 1, indicating an acceleration in the corrosion process. In the middle (3 to 10 days) and later periods (10 to 40 days), the value of n is less than 1, suggesting a deceleration in the corrosion process. This deceleration occurs due to the formation of corrosion products on the surface of GI-coated steel, which inhibits the corrosion process. For ZM-coated steel (Figure 3c), the corrosion process can also be divided into two periods. In the first period of exposure (1 to 10 days), the value of n is greater than 1, indicating an acceleration in the corrosion process of ZM-coated steel. In the second period (10 to 40 days), the value of n is less than 1, suggesting that the decreased corrosion process should be attributed to the formation of corrosion products. By comparing the weight loss of the two types of steels, it is observed that ZM-coated steel has a weight loss of approximately 5% compared to the weight loss experienced by GI-coated steel after being exposed to a salt spray for 40 days. These results further validate the influence of the new phase structure of ZM-coated steel on the corrosion performance of galvanized steel.

#### 3.2.3. Evolution of the Coated Steel Structure and Composition

**Figure 4:**
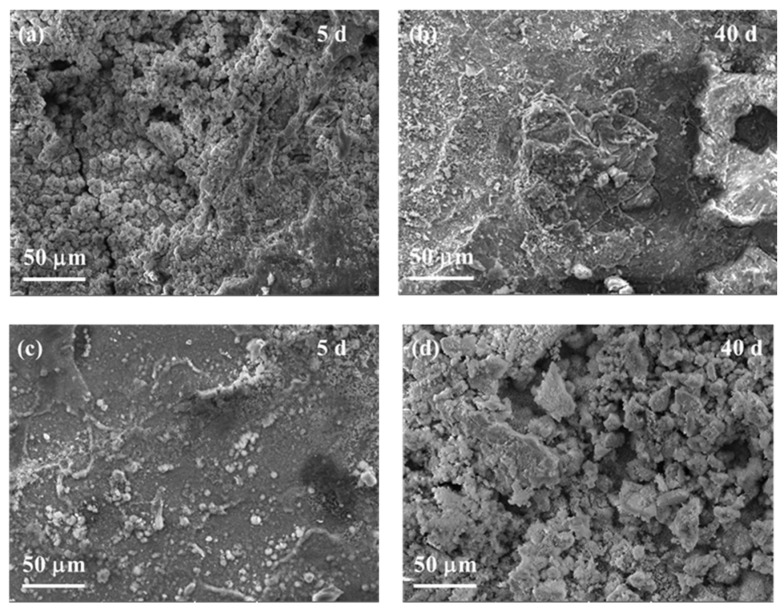
Morphologies of the surface of GI- (**a**,**b**) and ZM-coated steel (**c**,**d**) after 5 days and 40 days NNST experiments.

The morphologies of corrosion products formed on the surface of GI- and ZM-coated steel after NNST are shown in Figure 4. After 5 days of NSST, the surface of GI-coated steel was covered by loosened corrosion products. An obvious corrosion pore could easily be observed on the surface of GI-coated steel after 40 days of NSST, as shown in Figure 4a,b. There were few products formed on the surface of ZM-coated steel after 5 days of NSST (Figure 4c). Many corrosion products were observed on the surface of ZM-coated steel after 40 days of NSST (Figure 4d).

EDS was utilized to analyze the composition of corrosion products on the surface of GI- and ZM-coated steel after NNST, with the findings presented in Table 3. For GI-coated steel, after 5 days of NSST, the zinc content was 49.68 wt.% with trace amounts of iron also detected in the corrosion products. This suggests that the aggressive agents penetrated the Zn coating and reached the Zn/Fe interface, causing corrosion of the underlying steel. After 40 days of NSST, only 2.83 wt.% of Zn remained, while the Fe content dramatically increased to 64.84%. These results indicate that the Zn coating had almost entirely corroded, resulting in serious corrosion of the underlying steel. In the case of ZM-coated steel, only a small amount of iron was detected after 40 days of NSST, and no red rust was observed throughout the entire NSST process (Figure 2); the content of O and Zn was 32.39 wt.% and 43.74 wt.%, respectively. These findings suggest that the corrosion product on ZM-coated steel mainly consisted of zinc compounds.

**Figure 5:**
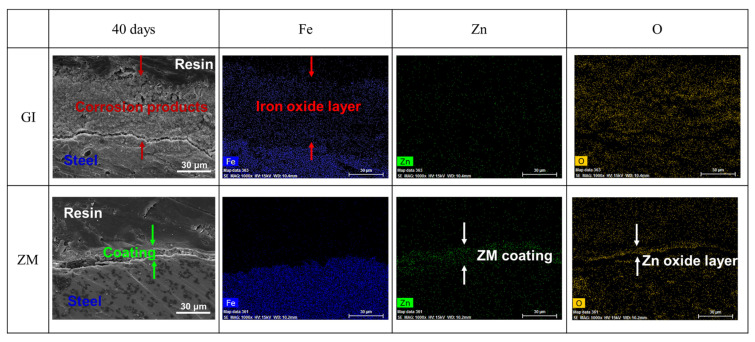
Cross-section images and elemental mappings of GI- and ZM-coated steel after 40 days of NSST experiments.

Cross-section images and elemental mappings of GI- and ZM-coated steel after 40 days of NSST experiments are shown in Figure 5. For GI-coated steel, the Zn element disappeared in Zn elemental mapping, indicating the GI coating was almost corroded. A 50 μm film was observed on the substrate which should be attributed to the aggressive media corroding the substrate to form a corrosion product film consisting of Fe and O, as shown in the Fe and O elements mappings. For ZM-coated steel, a 20 μm film was observed on the substrate, slightly larger than on the ZM-coated steel before the NSST experiment. A Zn accumulation layer was observed with a thickness of 20 μm, coupled with the accumulation of O but without Fe. These results indicated the ZM coating reacted with aggressive media to form Zn compounds. The aggressive media did not penetrate the ZM film to corrode the substrate. The cross-section results are consistent with the surface images and EDS results as shown in Figure 4 and Table 3, respectively.

**Figure 6:**
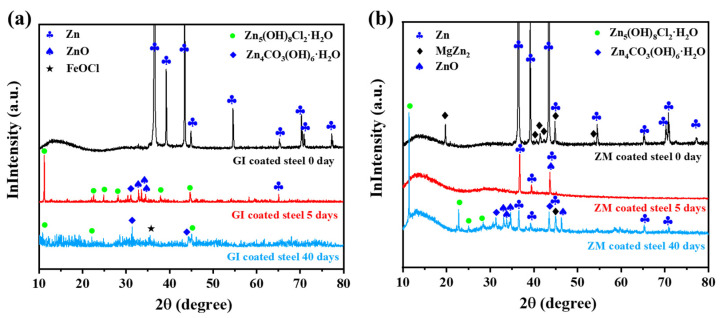
XRD patterns of corrosion products of (**a**) GI- and (**b**) ZM-coated steel after 5 d and 40 d exposure to salt spray test.

XRD analysis was performed to identify the corrosion products formed on GI- and ZM-coated steel after the NSST experiment. Figure 6a shows the XRD patterns of GI-coated steel after NSST. After 5 days of NSST, new peaks corresponding to ZnO, Zn_5_(OH)_8_Cl_2_·H_2_O, and Zn_4_CO_3_(OH)_6_·H_2_O were observed [30,31]. These findings indicate that the Zn coating reacted with the aggressive species (Cl^−^) and dissolved species (O_2_, CO_2_) present in the salt spray. After 40 days of NSST, a new peak correspondence to FeOCl emerged, suggesting the aggressive agents penetrated the Zn coating to the Zn/Fe interface and caused the corrosion of substrate steel, which is consistent with the rust products observed on the GI-coated steel surface (Figure 2) [31]. The absence of FeOCl in the 5 days of the NSST sample was probably attributed to the limited formation of this compound. Figure 6b shows the XRD patterns of ZM-coated steel after NSST. After 5 days of NSST, the peaks corresponding to MgZn_2_ disappeared and the intensity of the Zn peak decreased, indicating corrosion of the ZM coating and formation of ZnO species on the ZM-coated steel. Until 40 days of NSST, the peaks corresponding to Zn_5_(OH)_8_Cl_2_·H_2_O and Zn_4_CO_3_(OH)_6_·H_2_O were observed on ZM-coated steel. The different compositions of the corrosion products formed on GI- and ZM-coated steel indicated different corrosion mechanisms for these two types of steel.

### 3.3. Corrosion Behavior of ZM- and GI-Coated Steel

The corrosion processing of GI- and ZM-coated steels in 3.5 wt.% NaCl solution was monitored using electrochemical methods, including electrochemical impedance spectroscopy, polarization, and SVET.

#### 3.3.1. Electrochemical Impedance Spectroscopy

**Figure 7:**
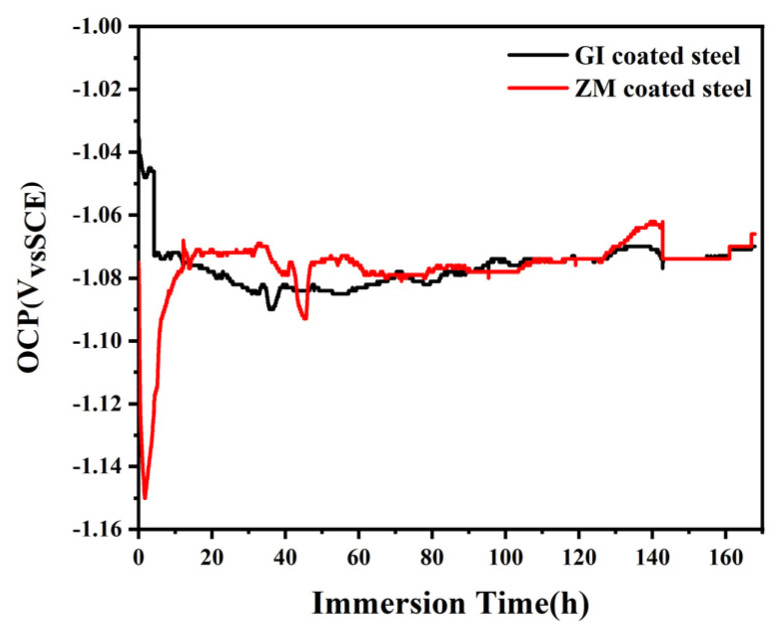
OCP of ZM and GI coated steel as a function of immersion time in 3.5 wt.% NaCl solution.

Electrochemical impedance spectroscopy of GI- and ZM-coated steel was performed under the open circuit potential (OCP) in 3.5 wt.% NaCl solution. Figure 7 shows the OCP of GI- and ZM-coated steel as a function of immersion time in 3.5 wt.% NaCl solution. The results show that the OCP values of GI-coated steel dramatically decreased at the initial stage, which might be attributed to the active dissolution of the Zn-rich phase, and then reached a relatively stable potential with the formation of corrosion products formed on the GI-coating surface after 5 h. The OCP values of ZM-coated steel rapidly decreased and were much lower than those of GI-coated steel because the potentials of Al and Mg_2_Zn are more negative than those of Zn [15]. Then, the potential increased rapidly and reached a similar potential as GI-coated steel.

**Figure 8:**
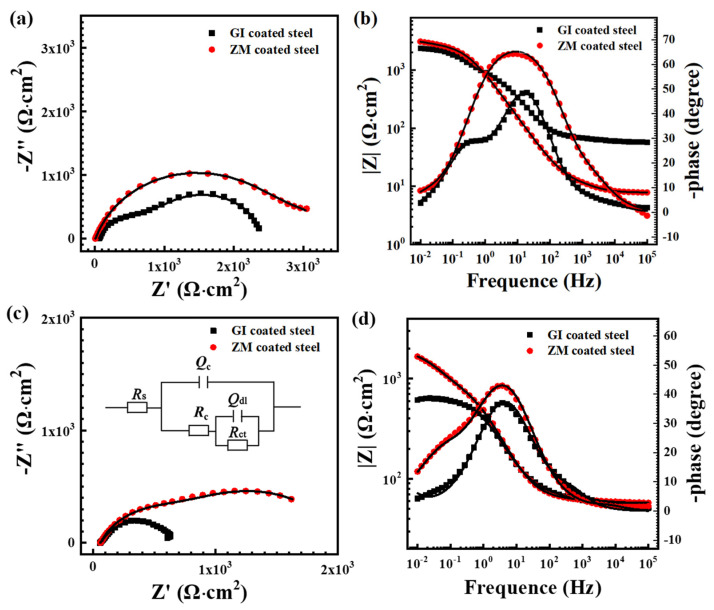
EIS spectroscopy of GI- and ZM-coated steels after 0.5 h (**a**,**b**), 168 h (**c**,**d**), open circuit potential (OCP) immersion in 3.5 wt.% NaCl solution (**c**), and (**d**) equivalent circuit diagrams were used to analyze the EIS data.

The electrochemical impedance spectroscopy results for GI- and ZM-coated steel immersed in 3.5 wt.% NaCl solution, as well as the equivalent circuit diagram used to analyze the EIS data, are illustrated in Figure 8. In Figure 8a,b, at the beginning of 0.5 h, the low-frequency impedance modulus (at 0.01 Hz) of ZM-coated steel is higher than that of GI-coated steel. The Nyquist diagram exhibits two capacitive reactance arcs, which can be attributed to the coating and the electrochemical corrosion reaction at the interface between solution and coating. The capacitive reactance arc radius of ZM-coated steel is larger than that of GI-coated steel, and the phase angle of ZM-coated steel is higher than that of GI-coated steel at the middle frequency of the Bode diagram. These results indicate an enhancement in the corrosion resistance of ZM-coated steel with the addition of Mg and Al. One probable interpretation is that the presence of Mg and Al elements in the molten bath promotes the formation of MgZn_2_ alloy phases through eutectic reactions, resulting in the development of binary or ternary eutectic phases with Al and Zn. This optimization of the phase structure and distribution within the ZM coating ultimately improves the corrosion resistance of ZM-coated steel.

When the immersion time increased to 168 h, the low-frequency impedance modulus (at 0.01 Hz) of GI-coated steel decreased dramatically from 2369 to 626 Ω∙cm^2^ but did not change too much for ZM-coated steel. This suggests that ZM-coated steel has higher long-term corrosion resistance. The phase constant of both GI and ZM-coated steel reduced, indicating that the coatings reacted with the NaCl solution, leading to the formation of corrosion products that altered the composition of the coatings.

The impedance data was fitted using the equivalent circuit model shown in Figure 8c. R_s_ represents the solution resistance; R_c_ and Q_c_ represent the coating resistance and capacitance, respectively; R_ct_ and Q_dl_ are the charge transfer resistance and double-layer capacitance, respectively [32]. The fitting data of GI- and ZM-coated steel after immersion in 3.5 wt.%NaCl solution for 0.5 h and 168 h are presented in Table 4.

In the initial immersion of 0.5 h, R_ct_ of ZM-coated steel (3580 Ω cm^2^) is significantly higher than that of GI-coated steel (1327 Ω cm^2^), suggesting the electrochemical reactions are more difficult to occur on ZM-coated steel. After immersion for 168 h, R_c_ and R_ct_ of GI-coated steel decreased to 113 Ω cm^2^ and 491 Ω cm^2^, respectively. Meanwhile, R_c_ and R_ct_ of ZM-coated steel decreased to 945 Ω cm^2^ and 1005 Ω cm^2^, respectively, which were much higher than those of GI-coated steel. These results suggest that the formation of a new phase in the ZM coating optimizes the coating structure, increases the coating resistance of the ZM coating, and exhibits a higher long-term corrosion protection property compared to GI-coated steel.

#### 3.3.2. Polarization Curves

**Figure 9:**
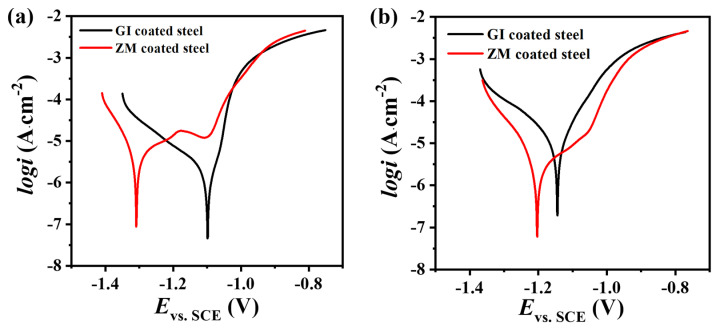
Polarization curves of GI and ZM after 0.5 h and 168 h immersion in 3.5 wt.%NaCl solution. (**a**) 0.5 h, (**b**) 168 h.

Potentiodynamic polarization measurement has been widely used to evaluate the anti-corrosion performances of metal and coated metals [33,34]. Figure 9 illustrates the polarization curves of GI- and ZM-coated steel after immersing them in 3.5 wt.% NaCl solution for 0.5 h and 168 h. GI-coated steel displayed active corrosion behavior during the initial 0.5 h immersion, with a corrosion potential (*E*_corr_) of approximately −1.1 V vs. SCE and a corrosion current (*i*_corr_) of 4.09 × 10^−7^ A∙cm^−2^. In comparison, ZM-coated steel exhibited a more negative *E*_corr_ than that of GI-coated steel (as shown in Figure 8a and Table 5), which might be attributed to the addition of Al and Mg forming a more active MgZn_2_ phase. At the beginning of the immersion, ZM-coated steel showed a relatively higher *i*_corr_ (1.27 × 10^−6^ A∙cm^−2^) compared to GI-coated steel. However, the presence of the MgZn_2_ phase distributed in the ZM coating also induced passivation in the anodic region (Figure 8a). After 168 h of immersion, *E*_corr_ for GI-coated steel decreased to −1.31 V vs. SCE, while *i*_corr_ increased to 8.47 × 10^−7^ A∙cm^−2^, indicating accelerated corrosion for GI-coated steel with prolonged immersion. However, in contrast, for ZM-coated steel, *E*_corr_ shifted to a more positive potential at −1.20 V vs. SCE, while *i*_corr_ decreased to 1.59 × 10^−7^ A∙cm^−2^, suggesting a corrosion inhibition for ZM-coated steel. In the 168 h immersion test, ZM-coated steel exhibited superior corrosion resistance compared to GI-coated steel, which was consistent with the findings of the electrochemical impedance spectroscopy test.

The above results indicate that the formation of the MgZn_2_ phase may affect the distribution of potential on the surface of the coating, potentially impacting the corrosion behaviors of the ZM-coated steels. The scanning vibrating electrode technique (SVET) was employed to investigate the potential distribution and current in the local area of coated steels.

#### 3.3.3. SVET Measurement

Distribution of corrosion current density calculated from the local corrosion potentials of GI- and ZM-coated steel during 0~168 h of immersion in 3.5 wt.% NaCl, obtained by SVET, according to the following calculation formula [20,21]:

(3) $$j=-\frac{\kappa ∆V}{A}$$

where j is the corrosion current density (A∙m^2^), κ is the conductivity of the electrolyte solution (S∙m), ∆V is the measured self-corrosion potential (V), and A is the amplitude of the vibrating electrode (m).

**Figure 10:**
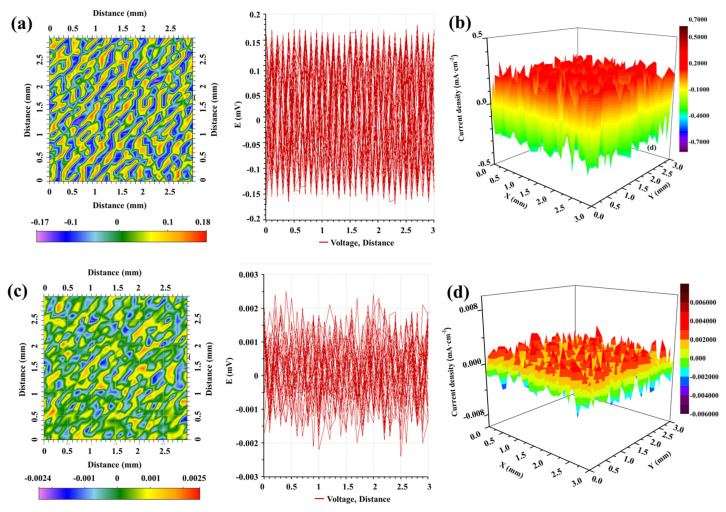
The distribution of potential and current density on the surface of GI-coated steel (**a**,**b**) and ZM-coated steel (**c**,**d**) at the beginning of immersion in 3.5 wt.% NaCl solution obtained from SVET.

Figure 10 describes the distribution of potential and current density on the surface of GI- and ZM-coated steel at the start of immersion in 3.5 wt.% NaCl solution. For GI-coated steel, the potential difference is around ±0.18 mV (Figure 10a). The red zone represents the anodic region (below 0.3 mA cm^−2^), while the blue–violet zone represents the cathodic region (above −0.3 mA cm^−2^) (Figure 10b). In the case of ZM-coated steel, the potential difference is around ±0.0025 mV (Figure 10c), which is three orders of magnitude lower compared to GI-coated steel. The anodic and cathodic current density is distributed between 0.004 mA cm^−2^ and −0.004 mA cm^−2^. These results indicate that the presence of MgZn_2_ increased the probability of thermodynamic corrosion, but it also refined the structure, reduced the potential difference in the anodic and cathodic regions on the ZM coating surface, and, consequently, decreased the galvanic corrosion. A previous study also shows the Zn-Mg phase is more corrosion-resistant during the corrosion process because the Zn-Mg phase oxidizes first, producing a passivation effect [35].

**Figure 11:**
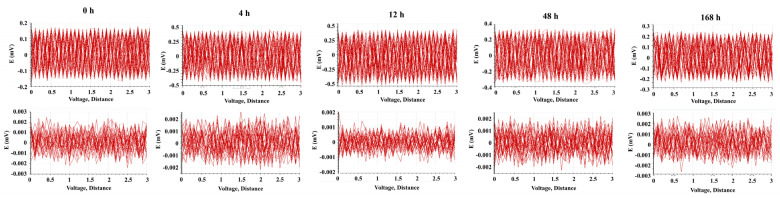
Potential distribution on the surface of GI-coated steel (**upper**) and ZM-coated steel (**bottom**) as a function of immersion time in 3.5 wt.% NaCl solution obtained from SVET.

The potential distribution and current density distribution on the surface of GI-coated steel and ZM-coated steel were measured as a function of immersion time in 3.5 wt.% NaCl solution using SVET, shown in Figure 10 and Figure 11, respectively. The potential distribution of GI-coated steel between anodic and cathodic increased from less than ±0.2 mV at 0 h to ±0.4 mV at 4 h, and then gradually decreased to ±0.25 mV at 168 h. The potential distribution of ZM-coated steel between anodic and cathodic decreased from less than ±0.0025 mV at 0 h to ±0.0015 mV at 12 h, and then gradually increased to ±0.0025 mV at 168 h.

**Figure 12:**
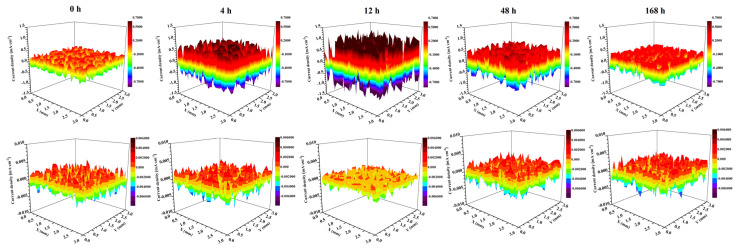
Current density distribution on the surface of GI-coated steel (**upper**) and ZM-coated steel (**bottom**) as a function of immersion time in 3.5 wt.% NaCl solution obtained from SVET.

The maximum value of the current density for GI-coated steel is 0.32 mA cm^2^ at 0 h, which increased to 1.39 mA cm^2^ at 12 h, and then decreased to 0.35 mA cm^2^ at 168 h (Figure 12). For ZM-coated steel, the maximum value of current density initially decreased from 0.0034 mA cm^2^ at 0 h to 0.0018 mA cm^2^ at 12 h and then gradually increased to 0.0032 mA cm^2^ at 168 h.

Comparing these two galvanized steels, the difference in potential and current density between cathodic and anodic sites is nearly three magnitude orders of GI-coated steel more than that of ZM-coated steel. One possible interpretation for this difference is that the injection of Al and Mg into the molten bath during the preparation of the coated steel facilitates the formation of the MgZn_2_ phase. This phase minimized the distribution of potential differences in the coating, thereby preventing galvanic corrosion. These results demonstrated that SVET is an effective methodology for detecting the anodic and cathodic evolution of galvanized steel, providing valuable information on galvanic corrosion.

## 4. Conclusions

In this work, a new ZM-coated steel was prepared by the addition of 1.5 wt.% Mg and 2 wt.% Al in the molten Zn bath throughout an industry hot-dip process. The composition, structure, and corrosion performance were system investigated. The main results were as follows:

1. The addition of Mg and Al elements to the molten Zn bath results in the formation of MgZn_2_ alloy phases via eutectic reactions. This process generates binary eutectic (Zn/MgZn_2_) and ternary eutectic (Zn/Al/MgZn_2_).
2. Polarization and electrochemical impedance spectroscopy results show that, after 168 h immersion in 3.5 wt.% NaCl solution, ZM-coated steel has a lower corrosion current, higher coating resistance (R_c_), and charge transfer resistance (R_ct_) compared to GI-coated steel. The neutral salt spray test also demonstrated that ZM-coated steel exhibited a higher long-term corrosion resistance compared to GI-coated steel.
3. SVET can distinguish anodic and cathodic sites on galvanized steel, providing precious local electrochemical information, i.e., the distribution of potential and current density on the surface. The presence of MgZn_2_ in the ZM coating refines the phase structure and improves uniformity, resulting in a low potential difference between the anodic and cathodic sites, and then the effective suppression of galvanic corrosion.

## Figures and Tables

**Table 1:** Thickness and average weight of the coatings.

Coatings	Thickness (μm)	Average Weight of Coating (g∙m^−2^)
Zn-Al-Mg (ZM)	13.7	87
Conventional galvanized (GI)	16.1	104

**Table 2:** Chemical compositions of ZM and GI.

Label	Site	Chemical Compositions (wt.%)					
		C	O	Mg	Al	Fe	Zn
ZM	I	6.03	1.37	1.07	0.88	0.28	90.37
	II	14.19	3.01	4.53	1.17	0.68	76.42
	III	7.05	2.52	3.79	4.68	0.72	81.24
GI	I	3.80	2.55	0.00	0.12	0.27	93.26
	II	7.66	4.32	0.00	0.15	0.85	87.02

**Table 3:** Chemical compositions of GI- and ZM-coated steel surface after 5 days and 40 days NSST experiments.

Lable	Time (d)	Chemical Compositions (wt.%)								
		C	N	O	Na	Mg	Al	Cl	Fe	Zn
GI	5	8.19	2.78	26.94	4.12	0.00	0.00	6.34	1.92	49.68
	40	4.94	1.98	25.12	0.00	0.00	0.00	0.09	64.84	2.83
ZM	5	23.18	4.72	30.01	0.00	0.25	0.11	1.11	0.77	39.63
	40	10.40	2.82	32.39	6.03	0.00	0.00	1.90	1.99	43.74

**Table 4:** Fitting data obtained from EIS of GI- and ZM-coated steel after 0.5 h and 168 h immersion in 3.5 wt.%NaCl solution.

Coating	Time (h)	R_s_ (Ω·cm^2^)	Q_c_ (Ω^−1^·cm^−2^·s^n^)	n_1_	R_c_ (Ω·cm^2^)	Q_dl_ (Ω^−1^·cm^−2^·s^n^)	n_2_	R_ct_ (Ω·cm^2^)
GI	0.5	61	1.15 × 10^−4^	0.78	1049	7.89 × 10^−4^	0.87	1327
	168	57	4.20 × 10^−4^	0.67	113	7.97 × 10^−5^	0.95	491
ZM	0.5	30	2.34 × 10^−5^	0.81	1098	1.68 × 10^−5^	0.58	3580
	168	59	4.46 × 10^−4^	0.72	945	3.56 × 10^−4^	0.74	1005

**Table 5:** Fitting parameters obtained from polarization curves of GI and ZM after 0.5 h and 168 h immersion in 3.5 wt.%NaCl solution.

Coating	Time (h)	*E*_corr_ (V vs. SCE)	*i*_corr_ (A∙cm^−2^)
GI-coated steel	0.5	−1.10	4.09 × 10^−7^
	168	−1.14	8.47 × 10^−7^
ZM-coated steel	0.5	−1.31	1.27 × 10^−6^
	168	−1.20	1.59 × 10^−7^

## Data Availability

The raw/processed data required to reproduce these findings can be obtained when contacting the corresponding authors.
